# AT101 exerts a synergetic efficacy in gastric cancer patients with 5-FU based treatment through promoting apoptosis and autophagy

**DOI:** 10.18632/oncotarget.9119

**Published:** 2016-04-30

**Authors:** Xi Wei, Wei Duan, Ying Li, Sheng Zhang, Xiaojie Xin, Lei Sun, Ming Gao, Qing Li, Dong Wang

**Affiliations:** ^1^ Department of Diagnostic and Therapeutic Ultrasonography, Tianjin Medical University Cancer Institute and Hospital, National Clinical Research Center of Cancer, Key Laboratory of Cancer Prevention and Therapy, Tianjin, China; ^2^ Cancer Center, Daping Hospital and Research Institute of Surgery, Third Military Medical University, Chongqing, China; ^3^ The Third Department of Breast Cancer, Tianjin Medical University Cancer Institute and Hospital, National Clinical Research Center of Cancer, Key Laboratory of Cancer Prevention and Therapy, Tianjin, China; ^4^ Department of Biochemistry and Molecular Biology, Tianjin Medical University Cancer Institute and Hospital, Tianjin, China; ^5^ Department of Thyroid and Cervical Tumor, Tianjin Medical University Cancer Institute and Hospital, National Clinical Research Center of Cancer, Key Laboratory of Cancer Prevention and Therapy, Tianjin, China

**Keywords:** gastric cancer, AT101, APE1, 5-FU, Her-2 positive

## Abstract

Gastric cancer remains a disease with a high mortality rate despite of multiple therapeutic strategies. So far, it is very important to develop new treatment approaches to improve current therapeutic efficacy in gastric cancer. Apurinic/apyrimidinic endonuclease (APE1) involves in DNA base excision repair (BER) during DNA damage pathway. APE1 was found to be associated with poor overall survival with gastric cancer patients. In the *in vitro* experiment, we tested APE1 inhibitor-AT101 could potently inhibit gastric cancer cell growth and further induce cancer cell apoptosis and autophagy through p53-dependent pathway. Downregulation of APE1 by AT101 has ability to suppress gastric cancer cell migration and renewal through inhibition of CD133, Nanog and LC3expression. Based on findings that Her-2 positive expression cases has poor prognosis from our dataset and TCGA database, we investigated the role of AT101 in synergetic efficacy with 5-FU treatment in Her-2 overexpression gastric cancer *in vivo*, indicating that AT101 is able to enhance 5-FU in the shrinkage of xenograft mice tumor and induction of cell apoptosis. In summary, the data obtained from our study showed APE1 is guided as a potential therapeutic target for gastric cancer. AT101 could be regarded as a potent inhibitor to promote chemotherapeutic sensitivity in patients with gastric cancer.

## INTRODUCTION

Gastric cancer is the main leading cause of cancer-related death around the world [1]. Advanced gastric cancer undergoes a poor prognosis with a median survival of less than 9 months [2]. The treatment plan within physician's options includes surgery, chemotherapy, radiation or other anticancer drugs. However, despite multiple therapeutic choices, the survival rate of patients with advanced gastric cancer remains poor in last several decades [3]. To facilitate optimal therapeutic strategies for gastric cancer is still a challenge for clinical application. Most of cytotoxicity agents for advanced cancer are related to induce genomic DNA damage. The inhibitors of DNA damage or DNA repair process appear to act as effective treatment for carcinomas [4].

Human apurinic/apyrimidinic endonuclease 1 (APE1) is an essential protein in regulation of the process of DNA base excision repair (BER) induced by DNA base damage [5]. APE1 is a globular α/β protein with N terminus for the redox activity and C terminus with DNA repair activity [5]. In human tumors, APE1 expression correlates with poor outcome or survival in variety of cancer patients [6]. As a target in cancer treatment, suppressing APE1 expression potentiates the activity of cytotoxic agents, enhancing chemotherapy sensitivity in cancer therapy [7]. AT101, as a BH3 mimetic and pan-BCL-2 inhibitor, contributes to potent potential anticancer role in several cancers [7, 8]. As a small molecular inhibitor of BCL-2 family member, AT101 induced apoptosis in human leukemic cells in a time- and dose-dependent fashion [9]. Previous study also showed AT101 revealed a synergistic efficacy with cisplatin to promote antitumor chemosensitivity in human non-small cells by inhibition of APE1/IL-6/STAT3 pathway [10]. Therefore, the hypothesis is raised that AT101, a potent inhibitor, would enable to enhance chemo-induced cell apoptosis and autophagy in gastric cancer cells.

Autophagy plays a critical role in evolutionarily conserved cellular degradation process, providing materials and energy for cell metabolism under certain stress, such as oncogenic stress or cancer drug stress [11]. The role of autophagy in chemoresistance should be under intense investigation. Thus, abrogation of autophagy would be beneficial to improve cancer chemosensitivity. And vice versa, autophagy is able to be a promising target for further cancer treatment. Apoptosis, a process of programmed cell death, has been targeted to overcome platinum resistance through enhancing its function in ovarian cancer [12]. Therefore, in our study, after investigation of patients with gastric cancer in a clinical cohort, we aimed to suppress APE1 expression to induce apoptosis and autophagy by using its inhibitor AT101 *in vitro*, leading to find out a novel therapeutic target for gastric cancer treatment.

## RESULTS

### APE1 positive expression associated with poor survival in patients with gastric cancer

**Figure 1:**
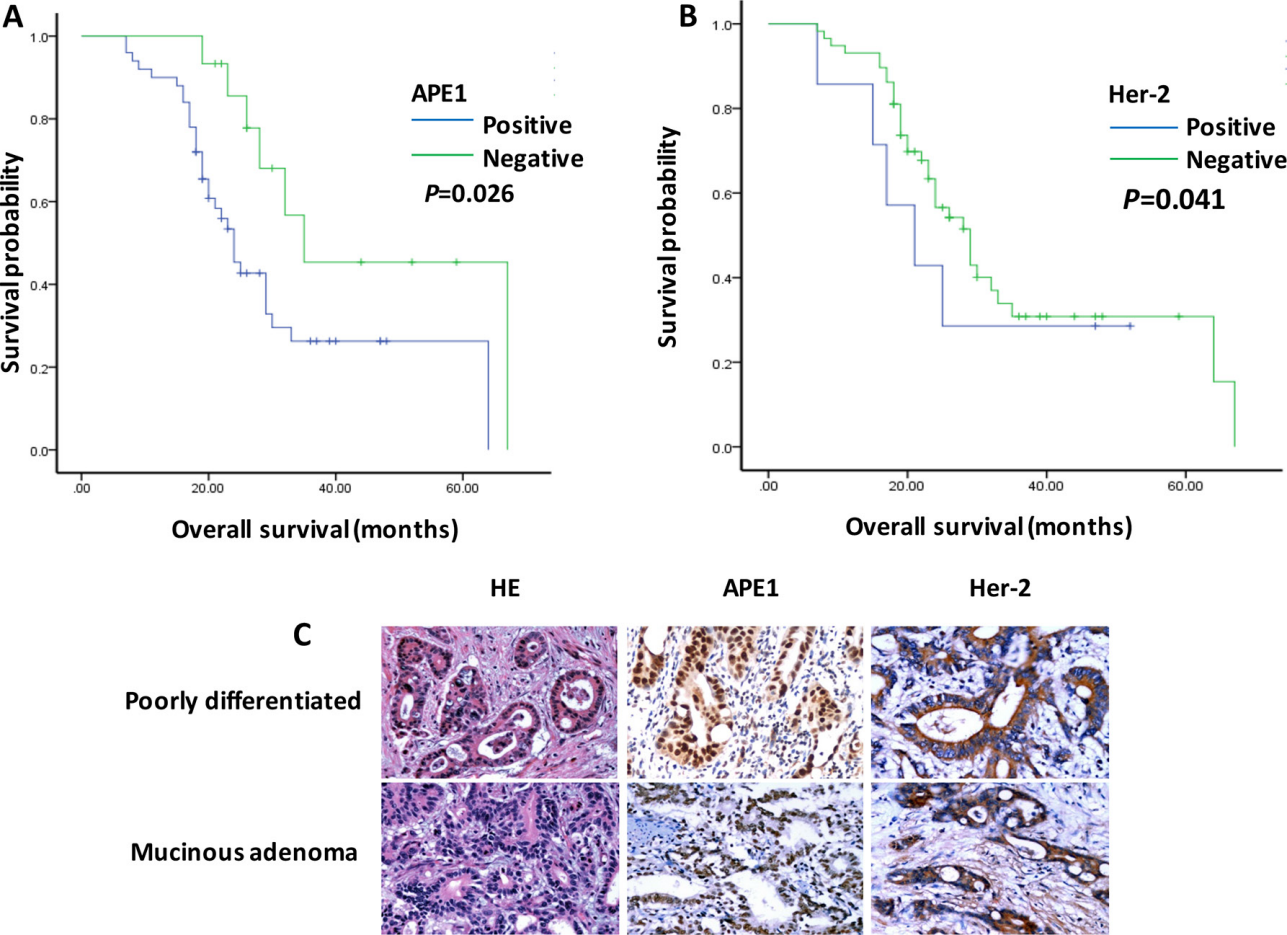
APE1 and Her-2 overexpression associated with poor prognosis of patients with gastric cancer (**A**) and (**B**) The Kaplan-Meier plot analyzed that APE1 and Her-2 positive expression associated with poor overall survival in patients with gastric cancer. (*t* test; *, *P* < 0.05) (**C**). The samples of patients with gastric adenocarcinoma were stained by Hemotoxylin & Eosin (HE) and immunohistochemistry. The expression of APE1 and Her-2 showed in the nucleus, cytoplasm and membrane (brown stain) in gastric cancer cells.

The total 65 patients with gastric adenocarcinoma were examined with APE1 expression with immunohistochemistry assay. The univariate and multivariate analyses showed that 50 cases with APE1 positive expression correlated with short overall survival in patients during follow-up in 24 months (*P* = 0.026 and 0.035) (Tables 1 and 2, Figure 1A and 1C). Additionally, we also found that Her-2 overexpression cases has poor prognosis consistent with results from previous study (*P* = 0.041 and 0.048) (Tables 1 and 2, Figure 1B and 1C) [16]. To some extent, APE1 and Her-2 overexpression associated with poor outcome of patients with gastric cancer, indicating potential markers for target therapy in clinical settings.

**Table 1:** The basic characteristics of patients with gastric cancer

Variables		Cases (%)
Total		65 (100)
Age	≥ 60	40 (61.5)
	< 60	25 (38.5)
Gender	male	47 (72.3)
	female	18 (27.7)
Stages	I	15 (23.0)
	II	12 (18.5)
	III	31 (47.7)
	IV	7 (10.8)
APE1	negative	15 (23.1)
	positive	50 (76.9)
Her-2	negative	58 (89.2)
	positive	7 (10.8)

**Table 2:** Association between the expression of APE1, Her-2 and overall survival in gastric patients

Factors		*N* (%)	Overall survival			
			MST(m)	*P*^a^	HR^b^ (95% CI)^b^	*P*^b^
APE1	Negative	15 (23.1)	35.0		1 (Reference)	
	Positive	50 (76.9)	24.0	0.026	2.57 (1.07–6.18)	0.035
Her-2	Negative	58 (89.2)	29.0		1 (Reference)	
	Positive	7 (10.8)	21.0	0.041	2.28 (1.16–4.49)	0.048

a Log-rank test.

b Estimated from the Multivariate Cox proportional hazards models.

N: Number; MST: median survival time; HR: Hazard ratio.

### AT101, as an inhibitor, contributes to gastric cancer cells suppression *in vitro*

**Figure 2:**
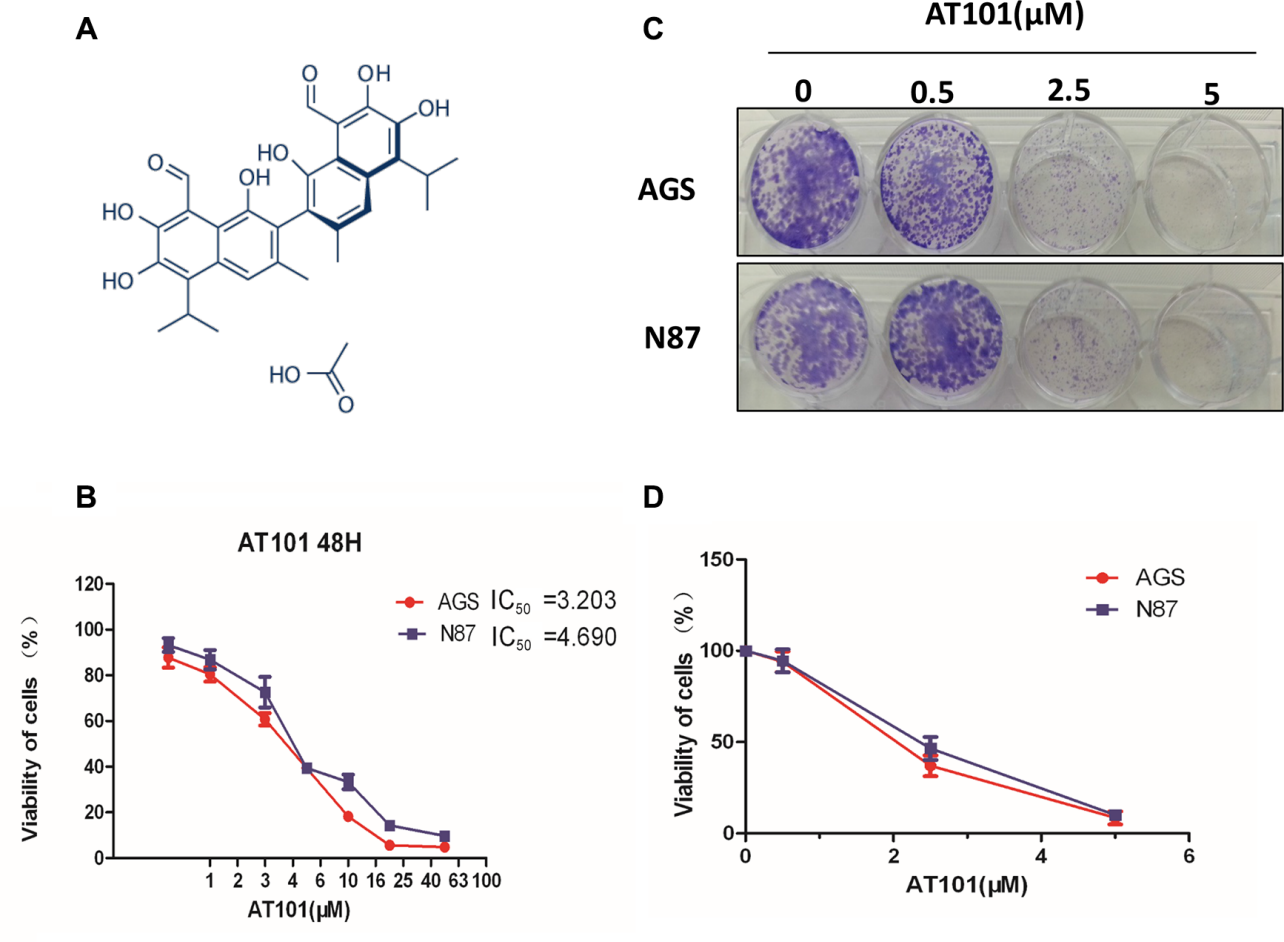
AT101, as an inhibitor, contributes to gastric cancer cells suppression *in vitro.* (**A**) Chemical structure of AT101 (**B**) The MTT assay was performed on the viability of AGS and NCI-N87 cells by AT101 at different concentrations (0.5–50 μM) and IC_50_ value was analyzed as indicated on the plot. (*t* test; *, *P* < 0.05). (**C** and **D**) Colony formation assays indicated that AT101 enables to inhibit AGS and NCI-N87 colonies formation (viability of cells) with increasing concentrations of AT101 (0–5 μM).

AT101, a promising anticancer agent, was reported in the inhibition of lung cancer cells proliferation and migration in our previous study [10]. To investigate the role of AT101 in the inhibition of gastric cancer cells, we tested two gastric cancer cells, AGS and NCI-N87, treating with different concentrations (0.5–50 μM) in MTT assays (Figure 2A and 2B). The data analysis indicated IC_50_ values in AGS and NCI-N87 cells were 3.2 and 4.6 treated by AT101, respectively. Furthermore, AT101 showed strong inhibitory effect on gastric cancer cells growth in the cell colony formation experiment (Figure 2C and 2D). These results indicated that AT101 could be a potent inhibitor in treatment of gastric cancer cells *in vitro*.

### Inhibition of APE1 by AT101 promotes apoptosis and autophagy of gastric cancer cells

**Figure 3:**
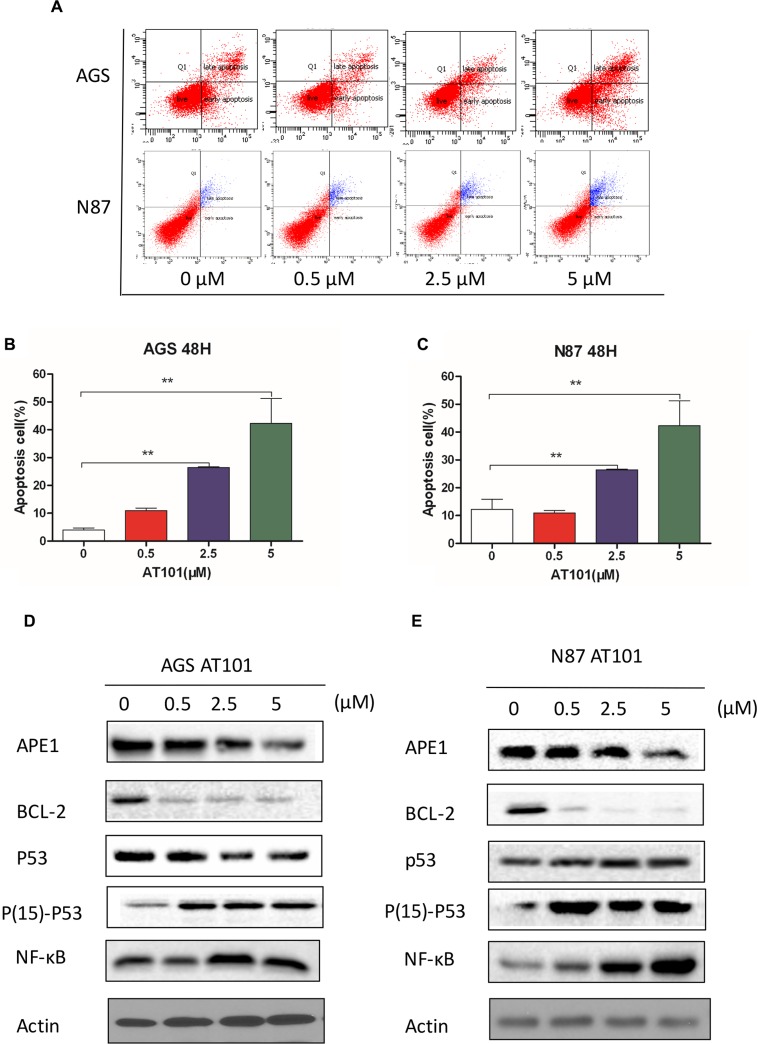

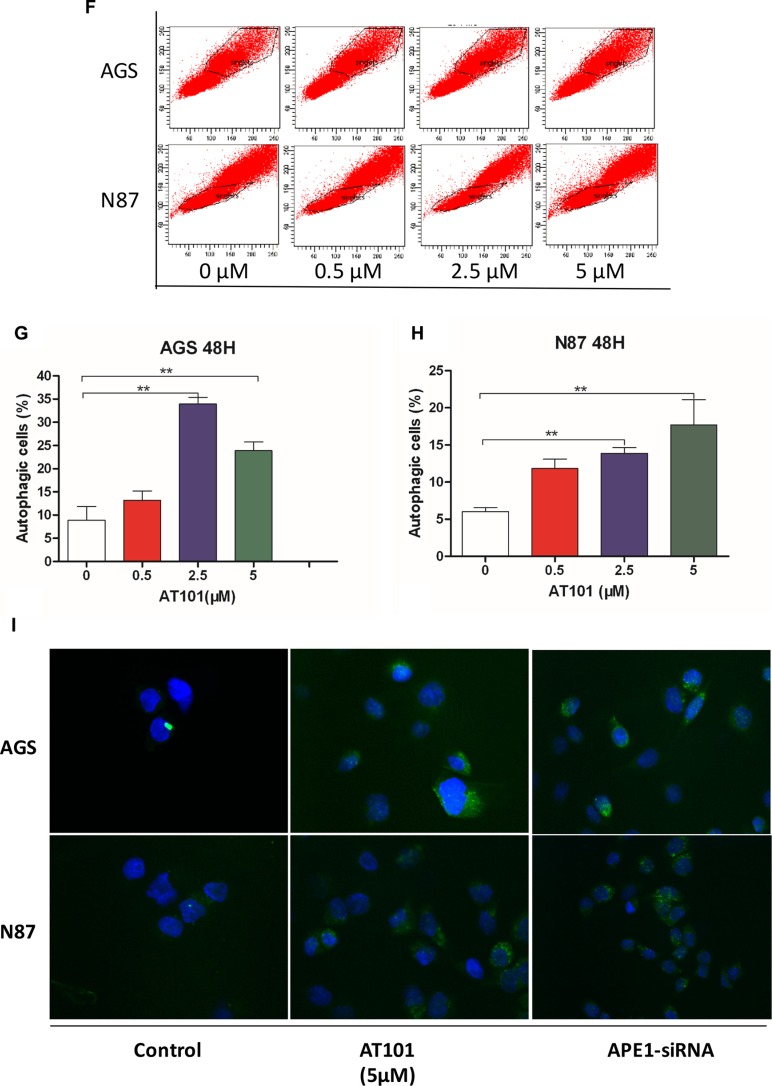
Inhibition of APE1 by AT101 promotes apoptosis and autophagy of gastric cancer cells AGS and NCI-N87 cells treated with AT101 at different concentrations of 0.5, 2.5, and 5 μM for 48 hours. (**A**–**C**) The apoptosis of cells was quantitated on the graph using the Annexin V: PE apoptosis detection kit and a flow cytometer. (*t* test; *, *P* < 0.05) (**D**, **E**) The markers of BCL-2, P53, Phosphate-activated P53 (Ser15) and NF-κB were detected by western blot assay. (**F**–**H**) The autophagic cells was detected by the Cyto-IDr autophagy detection kit and analyzed using the green (FL1) channel of the flow cytometer. (**I**) The cellular autophagy induced by the concentration of AT101 (5 μM) and APE1 siRNA was examined by confocal microscopy with the application of Cyto-IDr autophagy detection kit.

In our study, the *in vitro* assay using Annexin V probe, added additional evidence that AT101 induced apoptosis in two gastric cells with increasing concentrations (0–5 μM), suggesting that the apoptotic effect induced by AT101 was a dose-dependent relationship (Figure 3A–3C). To provide more evidence to this potential phenotype in two cell lines, we detected BCL-2, p53 and phosphated -p53, NF-κB markers with increasing dose of AT101 in western blot assay, indicating APE1 inhibitor has ability to decrease BCL-2 expression and to accelerate phosphate-p53 and NF-κB expression but without induction of p53 level (Figure 3D and 3E).

In order to illustrate the role of APE1 inhibition in autophagy of gastric cancer cells, we used Cyto-IDr fluorescent probe autophagy detection assay to exam autophagy cell markers in AGS and NCI-N87 cell lines (Figure 3I). The results showed that after inhibition of gastric cancer cells by AT101 (5 μM) or APE1 siRNA, both AGS and NCI-N87 demonstrated green autophagy dye gathered around cells, indicating APE1 suppression is able to induce autophagy in gastric cancer (Figure 3G and 3H). Moreover, the amount of autophagic cells increased according to dose accelerating of AT101 treatment using flow cytometry assay (Figure 3F). Taken together, AT101 appears to be a potent inhibitor of APE1 expression, facilitating gastric cancer cells apoptosis and autophagy *in vitro*.

### Suppression of APE1 enables to inhibit gastric cancer cells migration and primitive stem cell features

**Figure 4:**
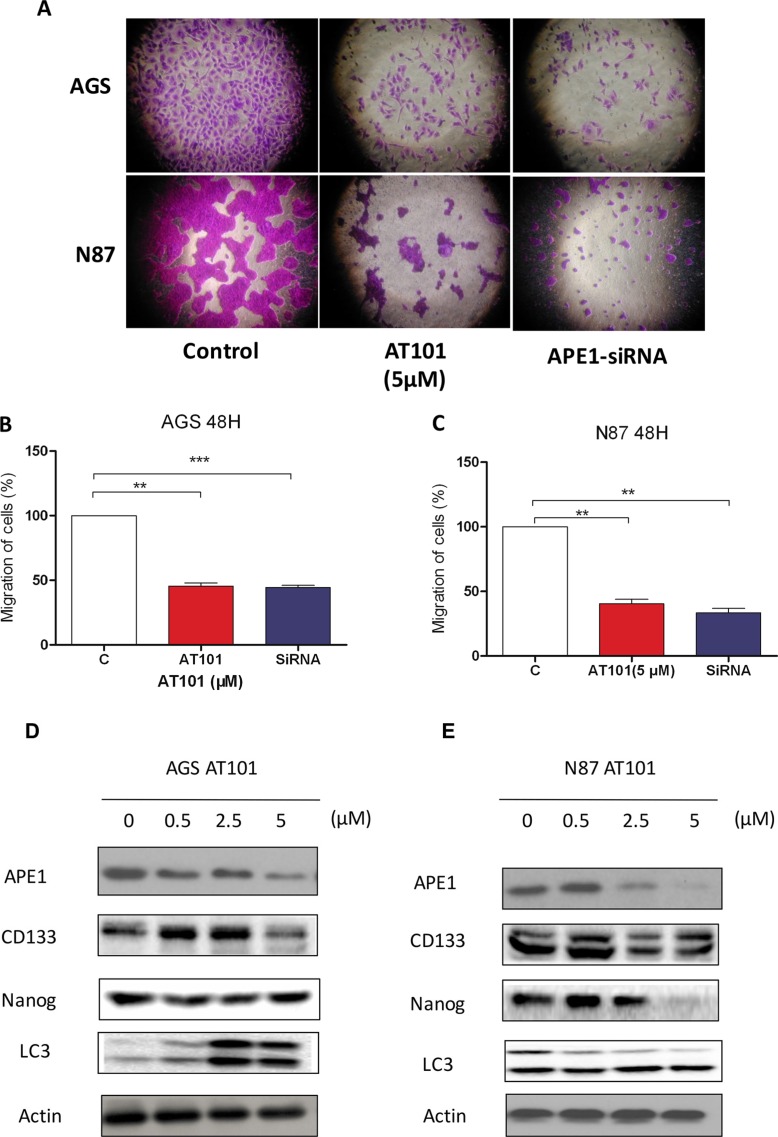
Suppression of APE1 enables to inhibition of gastric cancer cells migration and renewal features (**A**) AGS and NCI-N87 cells treated with the concentration of AT101 (5 μM) and APE1 siRNA for observing migration determined by transwell assays with matrigel. (**B**) and (**C**) The statistical data was shown on the plot (*t* test; *, *P* < 0.05). (**D**) and (**E**) Several stem-cell like markers (CD133, Nanog and LC3 (lower band shown)) was detected in AGS and NCI-N87 cells with incubation of AT101 (0.5, 2.5, and 5 μM) for 48 hours by western blot.

Whether inhibition of APE1 expression contributes to decrease gastric cancer cell migration is poorly understood. Using transwell assay to analyze AGS and NCI-N87 cell migration, the shown data displayed that percentage of migration of AGS and NCI-N87 were significantly decreased when treating with AT101 or APE1 siRNA (Figure 4A–4C). Then, further testing three indicators of stem cells (CD 133 or Siglec-3-sialic acid binding Ig-like lectin 3; Nanog and Microtubule-associated protein 1A/1B-light chain 3-LC3) in AGS and NCI-N87 cells, we found that inhibition of APE1 by increasing dose of AT101 facilitated the decline of CD133, Nanog and LC3 expression in western blot assay (Figure 4D and 4E). Overall, there is accumulating evidence that gastric cancer cell migration and stem cell like characteristic could be suppressed by APE1 inhibitor.

### The role of AT101 in the treatment of Her-2 positive gastric cancer with 5-FU based therapy *in vivo*

**Figure 5:**
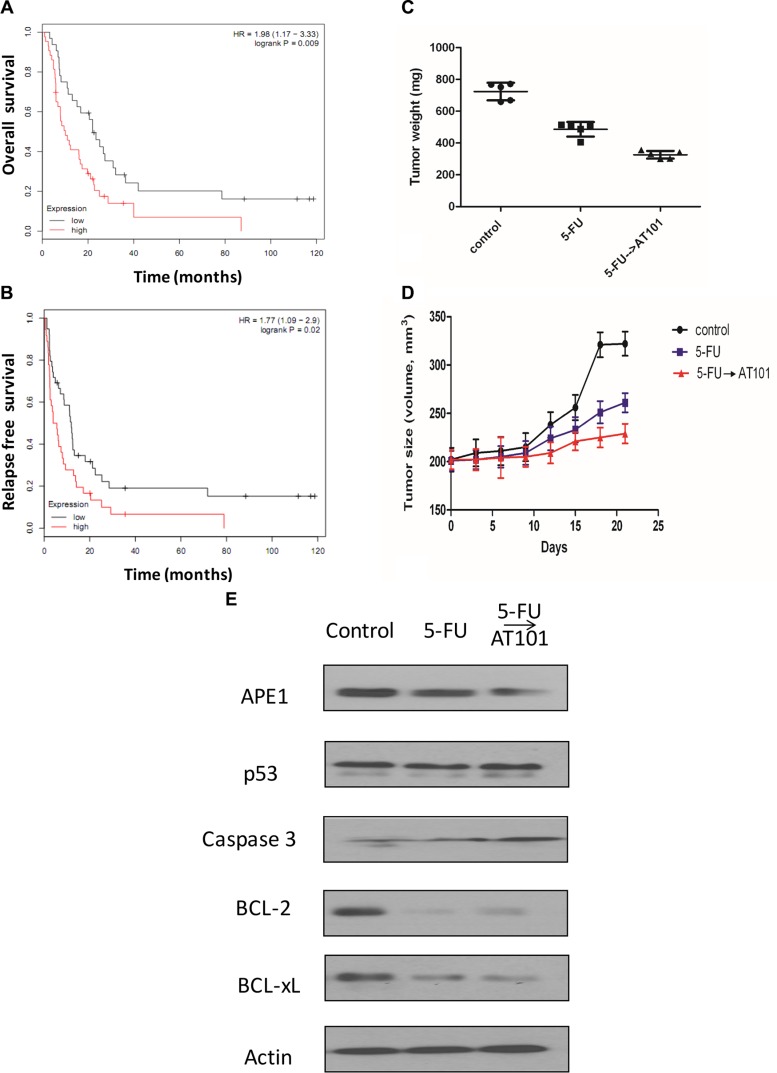
The role of AT101 in the treatment of Her-2 positive gastric cancer with 5-FU based therapy *in vivo* (**A**) Kaplan-Meier plot of 75 patients with Her-2 positive gastric cancer following 5-FU treatment showing low APE1 (*n* = 32) expression associated with shorter overall survival. (**B**) Kaplan-Meier plot of 75 patients with Her-2 positive gastric cancer following 5-FU treatment showing low APE1 (*n* = 39) expression associated with poorer relapse free survival. (**C**) and (**D**) The tumor weight and volume of xenograft mice model in 5-FU sequential treatment with AT101 became smaller than those with 5-FU treatment alone. (**E**) The downregulated of BCL-2 and Bcl-XL and increased caspase 3 expression were shown in a subset of tumors after 5-FU and 5-FU sequential AT101 treatment.

To further study the treatment potential of AT101 in gastric cancer, we first analyzed 75 patient groups with APE1 expression using quantile based on clinical information from The Cancer Genome Atlas (TCGA) dataset. Kaplan-Meier survival analysis showed APE1 positive expression associated with shorter overall and relapse free survival of seventy-five Her-2 positive patients with gastric cancer under 5-FU based treatment (logrank *P* = 0.009 and 0.02, respectively) (Figure 5A and 5B). In the *in vivo* experiment, Her-2 positive gastric cancer cell was used to establish a NCI-N87 xenograft mice model for further investigation. Comparing to 5-FU alone treatment *in vivo*, we found that the average weight of mice tumors with sequential-treatment on 5-FU following up AT101 decreased significantly (*P* < 0.05) (Figure 5C). The xenograft tumor size was also shrunk after treatment of 5-FU with APE1 inhibitor for 2 weeks, compared to control and 5-FU alone groups (Figure 5D). When testing apoptotic markers including p53, caspase 3, BCL-2 and BCL-xL, we revealed that increasing level of caspase 3 and decreasing trend of BCL-2 and Bcl-XL after treatment of 5-FU and sequential treatment of AT101 (Figure 5E). All of above results uncovered that 5-FU sequential treatment with AT101 in Her-2 positive gastric carcinomas has a potential inhibitory effect on xenograft tumors shrinking and apoptosis *in vivo*. Taken together, our accumulating data demonstrates that Her-2 positive gastric cancer patients could have benefit from the 5-FU sequential treatment with AT101, because of inhibition of tumor APE1 expression in the 5-FU based therapeutic strategy.

## DISCUSSION

Gastric cancer patients associate with poor prognosis worldwide, despite of conventional cancer therapies including surgery, radiation and chemotherapy. APE1 plays a central role in the DNA base excision pathway, obtaining as a redox co-activator of different transcription factors, such as nuclear factor-κB (NF-κB), hypoxia inducible factor-1 (HIF-1) and activator protein-1 (AP-1) in cell cycle system [17]. Previous studies found that genetic variant rs 1760944 and rs 3136820 in APE1 play a critical role in gastric cancer survival outcomes in Chinese population [18, 19]. In our study, APE1 positive expression was also tested to correlate with poor overall survival in Chinese patient cohort. Al-Attar et al. reported that APE1 expression associated with poor survival in patients with ovarian, gastro-oesophageal and pancreatic cancers, being considering as a potential drug target [20]. Multiple studies elucidated that as predictors of gastric cancer risk, APE1 ploymorphism and protein expression present a positive relationship with prognosis of gastric carcinoma [19–21].

Clinical studies provide mounting evidence that APE1 expression or subcellular dysregulation enhance sensitivity of tumors towards radio-or chemotherapy [22, 23]. As a potent APE1 inhibitor, AT101 was used to enhance sensitivity of platinum-based chemotherapy in non-small cell lung cancer treatment [8]. Thus, we propose to find out whether AT101 promote antitumor activity in gastric cancer treatment. According to our strategy, we first investigate IC_50_ of AT101 in treatment of gastric cancer cells *in vitro*, finding that AT101 has ability to inhibit viability of gastric cancer cells. The functional relationship between apoptosis and autophagy undergo crosslink during self-killing and self-eating process in cellular settings [24]. Inhibition of autophagy in tumor cells led to tumor regression and extended survival in cancer xenografts. However, other studies showed autophagy plays a harmful role in enhancing efficacy of anticancer drugs. In our study, we found AT101 promoted dose-dependent autophagy with presentation of autophagosome in gastric cancer cells. Our findings indicated that the increased apoptosis in AGS and NCI-N87 cell lines were accompanied with the accelerating dose of AT101 treatment, along with phosphated p53 and NF-κB level increasing and BCL-2 expression decreasing.

Furthermore, our study pointed out that dose-dependent AT101 displayed suppression of gastric cancer cell migration, meanwhile, decreasing cell renewal markers-CD133, Nanog and LC3 expression after inhibition of APE1 expression. In this case, down-regulation of APE1 by AT101 facilitates gastric cancer cell migration and self-renewal, further maybe involving in mechanism of gastric cancer metastasis in clinical application. 5-fluorouracil (5-FU) is the most widely used chemotherapy agent in the treatment of advanced gastric cancer. Her-2 positive in gastric cancer associated with poor prognosis, but other studies reported no significant relationship with pathological parameters or overall survival [25].

Although Her-2 positive expression accounts to low percentage (approximately 8%) of gastric patients, the survival of this specific patient's population could be beneficial from trastuzumab as a first molecule drug since 2010 [26]. The results of our clinical analysis showed that Her-2 overexpression patients associated with poor prognosis but limited number of cases was included. We still engaged to figure out whether both these two predicted markers have some relationship with gastric cancer patients with 5-FU based treatment after further analyzing TCGA dataset of 75 gastric patients with Her-2 positive based on 5-FU treatment, we found that APE1 positive expression correlated with poor overall and relapse-free survival. Further research in the gastric cancer xenograft mice model clarified our hypothesis that inhibition of APE1 expression by AT101 sequential with 5-FU results in shrinkage of tumor volume and cancer cell apoptosis. Accordingly, APE1 could be regarded as a potential target with synergistic antitumor effects of 5-FU in the treatment of gastric cancer patients.

In conclusion, we identified that APE1 as a critical oncogene associated with poor prognosis in gastric cancer patients. Being a potent inhibitor of APE1, AT101 involves in induction of apoptosis and autophagy of gastric cancer cells *in vitro*. Functionally, APE1 inhibitor suppresses gastric cancer cell migration and renewal to be synergetic effect on 5-FU based therapy in gastric cancer, providing clues for further clinical strategy decision making.

## MATERIALS AND METHODS

### Study population

The total of 65 patients with gastric cancer was enrolled in this study at Tianjin Medical University Cancer Institute and Hospital (Tianjin, China) and Daping Hospital, Third Military Medical University (Chongqing, China) between June 2009 and July 2011. All patients were diagnosed as adenocarcinoma with age ranges from 30 to 81 years old, and average was 60.2 years old. Using immunohistochemistry assay as described in previous studies [24–26], APE1 expression was measured in 65 cases. The median follow-up time was 28.5 months. The overall survival (OS) was analyzed from the date of surgery until either the time of death or the end of follow-up. This study was approved by the ethics committees of Tianjin Medical University Cancer Institute and Hospital and Daping Hospital.

### Cell lines and cell culture

AGS and NCI-N87 cells are human gastric cell lines that were obtained from ATCC (American Type Culture Collection) and were maintained in RPMI-1640 (Roswell Park Memorial Institute-1640) medium with 10% fetal bovine serum (Corning Cellgro Inc., Herndon, VA, USA). The cells were cultured at 37°C in a 5% CO_2_ incubator.

### Cell viability assay

AT101 (Selleckchem Inc, Shanghai, China) was dissolved in DMSO with a stock concentration of 50 mM, and was freshly diluted to the desired concentrations with culture medium. The final concentration of DMSO was at 0.05% (v/v). The MTT assay was performed to examine the effect of AT101 on the viability of AGS and NCI-N87 cells. Briefly, cells were seeded in 96-well culture plates at a density of 8 × 10^3^ cells/well. After attaching, we treated cells with AT101 at different concentrations (0.5–50 μM). The control cells received the vehicle only. After 24 hours incubation, 10 μL MTT (5 g/L) was added to each wells and cultured for 4 hours. Then, 100 μL DMSO was added in the plates. The absorbance at the 570 nm wavelength was measured with a Synergy H4 Hybrid microplate reader (BioTek Inc., Winooski, VT, USA). The IC_50_ values were analyzed using GraphPad Prism 6.0 (GraphPad Software, Inc., La Jolla, CA, USA).

### Colony formation assays

The AGS and N87 cells (6 × 10^4^/well) were plated in 6-well plates for 24 hours and incubated with AT101 at different concentrations of 0.5, 2.5, and 5 μM for 48 hours and allowed to grow until colonies are visible (8–10 days). Colonies were stained by crystal violet. The assays were performed in triplicate by the Quantity One software and were repeated three times.

### Quantification of cellular apoptosis

The effect of AT101 on apoptosis of AGS and NCI-N87 cells was quantitated using the Annexin V: PE apoptosis detection kit (San Jose, CA, USA). The cells were incubated with AT101 at different concentrations of 0.5, 2.5, and 5 μM for 48 hours. In separate experiments, cells were suspended, washed by PBS. The cells were resuspended at a concentration of 1 × 10^6^ cells/ml in binding buffer. A quota of cell suspension (100 μl) was transferred into a clean 5 ml tube and incubated with 5 μl of Annexin V: PE and 5 μl of 7-amino-actinomycin D (a vital nucleic acid dye) in the dark for 15 minutes at room temperature. A quota of binding buffer (400 μl) was then added to each tube, and the number of apoptotic cells was quantified using a flow cytometer within one hour.

### Quantification of cellular autophagy

To examine the effect of AT101 on autophagy in AGS and NCI-N87 cells, cellular autophagy was detected using flow cytometry. AGS and NCI-N87 cells were seeded in 60 mm dishes for 24 hours and then treated with AT101 (0.5, 2.5, and 5μM) for 48 hours. Following the treatment, digestion and centrifuge, and then, the cell pellet was washed with 1×assay buffer and in the Cyto-ID and Hoechst autophagy detection kit and resuspended in 250 μL fresh 1×assay buffer. Cells were analyzed using the green (FL1) channel of the flow cytometer.

### Confocal fluorescence microscopy

The effect of AT101 on cellular autophagy was further examined using confocal microscopy with the application of Cyto-IDr autophagy detection kit according to the manufacture's instruction. The AGS cells were seeded into 8-well chamber slide at 30% confluence. After incubation overnight, the cells were treated with AT101 at 0.5, 2.5, and 5 μM. After incubation for 24 hours, were washed with 1 × assay buffer, following by incubation with 100 μL of microscopy dual detection reagent for 30 minutes at 37°C in the dark. After incubation, the cells were washed with 1 × assay buffer to remove the detection reagent, and then examined using a TCS SP2 laser scanning confocal microscope (Leica, Wetzlar, Germany) using a standard fluorescein is thiocyanate filter set for imaging the autophagy signal at wavelengths of 405/488 nm.

### The transwell assay

Using Corning Matrigel invasion chamber (Biocoat, MA), cell migration assay was performed. Cells were seeded in 24-well plates at a density of 30,000 cells per well, exposing to scramble, APE1siRNAs (siRNA pools from Origene Company) and AT101. In this assay, the upper chamber was filled with medium without serum, while the lower chamber filled with 20% FBS medium. After 48 hours, cells were fixed with 4% formaldehyde and methanol. Then after scraping off non-migration cells with cotton swabs, the number of migrated cells was counted under an inverted microscope.

### Western blotting analysis

AGS and NCI-N87 cells were washed with pre-cold PBS after 24 hours treatment with AT101 at 0.5, 2.5, and 5 μM, lysed with the RIPA buffer (50 mmol HEPES at pH 7.5, 150 mmol NaCl, 10% glycerol, 1.5 mmol MgCl_2_, 1% Triton-× 100, 1 mmol EDTA at pH 8.0, 10 mmol sodium pyrophosphate, 10 mmol sodium fluoride) containing the protease and phosphatase inhibitor cocktails, and centrifuged at 3000 × g for 10 minutes at 4°C. Protein concentrations were measured using Pierce BCA protein assay kit. Equal amount of protein sample (30 μg) was resolved by sodium dodecyl sulfate polyacrylamide gel electrophoresis (SDS-PAGE) sample loading buffer and electrophoresed on 7%–12% SDS-PAGE mini-gel after thermal denaturation at 100°C for 5 min. Proteins were transferred onto PVDF membrane at 400 mA for 1–2 hour at 4°C. Membranes were probed with indicated primary antibody overnight at 4°C and then blotted with respective secondary antibody. Visualization was performed using Bio-Rad ChemiDocTM XRS system (Hercules, CA, USA) with enhanced-chemiluminescence substrate and the blots were analyzed using Image Lab 3.0 (BioRad, Hercules, CA, USA). Protein level was normalized to the matching densitometry values of the internal control β-actin. The antibody against human β-actin was from Santa Cruz Biotechnology Inc. (Dallas, TX, USA).

### TCGA data set

The clinical information of patients was downloaded from website of The Cancer Genome Atlas (http://cancergenome.nih.gov). After analyses of 75 gastric cancer patients with treatment of 5-FU separated by Her-2 expression, Kaplan-Meier survival curves were performed to test the significance of difference of survival. Data analysis and statistical manipulation were used R 3.2.1.

### Animal xenograft models

The animal experiment in xenograft mice models was approved by the Ethics Committee of the Third Military Medical University, Chongqing, China. Three groups (*n* = 5 per group) were assigned with 3-to-4 weeks old BALB/c nude mice. The NCI-N87 Her-2 positive cells were cultured at 90% confluence to prepare for a cell suspension in amount of 2.0 × 10^6^ cells/100 μL. After subcutaneously inoculating tumor cells in the left anterior axilla of nude mice, mice were treated with vehicle control (sesame oil), AT101 (dissolved in sesame oil, 35mg/kg/day) and 5-FU (4mg/kg/day) by oral gavage (vehicle and AT101) or intraperitoneal injection (5-FU) for 10 consecutive days.

### Statistical analysis

All of the data are presented as the mean ± standard deviation (SD). Comparisons of multiple groups were evaluated by one-way analysis of variance (ANOVA) followed by Tukey's multiple comparison procedure. *P* < 0.05 was considered statistically significant. All of the assays were performed at least three times independently.
